# No clinical benefit from gender-specific total knee replacement implants: a systematic review

**DOI:** 10.1051/sicotj/2020023

**Published:** 2020-07-03

**Authors:** Elliot Sappey-Marinier, John Swan, Cécile Batailler, Elvire Servien, Sébastien Lustig

**Affiliations:** 1 FIFA Medical Center of Excellence, Department of Orthopaedic Surgery and Sports Medicine, Croix-Rousse Hospital, Hospices Civils de Lyon 69004 Lyon France; 2 LIBM – EA 7424, Interuniversity Laboratory of Biology of Mobility, Claude Bernard Lyon 1 University 69100 Villeurbanne France; 3 Univ. Lyon, Claude Bernard Lyon 1 University, IFSTTAR, LBMC UMR_T9406 69622 Lyon France

**Keywords:** Total knee arthroplasty, Unisex design, Gender-specific design, Total knee replacement, Systematic review

## Abstract

*Introduction*: Total knee arthroplasty (TKA) remains the treatment of choice for severe osteoarthritis of the knee and nearly 60% of patients undergoing TKA are women. Females present three notable anatomic differences. Thus, gender-specific (GS) components were introduced to accommodate the females’ anatomic differences. No systematic review has been published since 2014. The aim of this study was to perform a recent systematic review of the literature to determine whether there is any clinical benefit of gender-specific implants compared to conventional unisex implants in total knee arthroplasty (TKA). *Methods*: This study included prospective randomized controlled trials (PRCTs) comparing clinical and radiological outcomes, and complications in TKA with gender-specific implants and conventional implants. All studies had a minimum follow-up of two years. *Results*: Three PRCTs published between 2010 and 2012 were included. These studies showed a low risk of bias and were of very high quality. We did not find superior clinical outcomes for gender-specific prostheses compared to conventional prostheses. However, gender-specific TKA reduced the number of patients with femoral component overhang compared to conventional TKA. *Conclusion*: In our systematic review, despite a lower overhang rate, gender-specific implants in female TKA showed no clinical benefit over standard unisex implants. Good clinical results with significant improvement were observed with both designs. There is a notable absence of new studies on this subject in recent years, and further research needs to be performed using various gender-specific implant designs to further define the role of gender-specific implants. *Level of evidence*: Systematic review, Level IV

## Introduction

Total knee arthroplasty (TKA) remains the treatment of choice for severe osteoarthritis of the knee [1] and nearly 60% of patients undergoing TKA are women [2–4]. Outcomes of TKA are influenced by several factors. Indeed, many discussions have focused on the effects of gender on the results of TKA [5–8]. Three notable anatomic differences in the females are well documented [9–12]. Women have a less prominent anterior condyle [13, 14], an increased quadriceps angle (*Q* angle) [15, 16], and a reduced mediolateral (ML)/anteroposterior (AP) aspect ratio [9, 17]. It has been emphasized that standard knee prostheses may not exactly match the native anatomy in female and male knees [18, 19]. This potential femoral component overhang may influence postoperative knee pain or reduce range of motion [10, 20, 21].

Thus, gender-specific (GS) components were introduced to address these issues. Instead of simply increasing the number of femoral implants with similar ML to AP ratios, the GS component is designed to better accommodate females’ anatomic differences with a narrower ML dimension for any given dimension. Moreover, to better match the native female anatomy the anterior flange thickness was reduced and the angle of the trochlear groove was increased [8, 22].

Two systematic reviews [23, 24] and meta-analysis were performed comparing clinical and radiographic results of TKA in female patients receiving standard unisex or GS prostheses. The authors of these two studies reported a lower femoral component overhang rate in the gender-specific group without any influence on clinical results. They concluded that gender-specific prostheses did not appear to confer any benefit in terms of clinician-reported and patient-reported outcomes for the female knee.

Thus, we decided to perform a recent systematic review of the literature in order to identify all new studies on this specific topic. We compared the clinical and radiographic outcomes of TKA in female patients receiving GS prostheses or standard unisex prostheses. Our outcome variables included clinical rating scores, radiological outcomes, or complications at a minimum follow-up of two years.

## Methods

### Literature search strategy

For this study, the Preferred Reporting Items for Systematic Reviews and Meta-Analyses (PRISMA) guidelines were followed [25]. A primary electronic search was performed using PubMed, Ovid Medline, and Cochrane library from their dates of inception to the 15th February 2020. To maximize search strategy sensitivity, the authors combined the terms “knee”, “arthroplasty”, “replacement”, “gender specific”, “conventional”, “standard”, and “unisex design” when searching in the title, abstract, keywords, and MeSH fields. A secondary search was performed examining the references cited in the articles found in the primary search. All articles were reviewed by two authors independently following this systematic approach. Each reviewer was blinded with regard to the determination of the other reviewer. Ethical approval was not necessary in this study as it only analyzed current studies and did not collect individual patient data. No external funding was received for this project.

### Selection criteria

Only prospective randomized controlled trials (PRCTs) comparing GS implants to conventional implants in TKA were included. All studies included a gender-specific implant group and a conventional or standard implant group with a minimum of 10 TKAs in each group and a minimum follow-up of two years. The authors included studies in the final analysis, if they reported clinical outcome scores, complications, or postoperative radiographic assessment. When several studies reported the results of the same patient series with different follow-ups, only the last study with the longest follow-up was analyzed. All publications included were limited to those written in the English language, involving human subjects and full-text availability for the articles. Reviews, duplicate studies, case reports, noncomparative studies, expert opinions, letters, and conference presentations were excluded.

### Data extraction

All the relevant data were extracted from article text, figures, and tables. Two investigators independently reviewed and extracted data from the retrieved articles. Discrepancies at the full-text stage were resolved by consensus between the two reviewers. If a consensus could not be reached, a third, more senior reviewer helped to resolve the discrepancy. The two independent reviewers collected information regarding the publication origin, publication date, authors, patient demographics (age, sample size, and body mass index (BMI)), prosthetic designs, and outcome measurements.

The primary outcomes were the clinical and radiological results. The clinical results included range of motion (ROM), the Knee Society score (KSS) [26], Hospital for special surgery (HSS) score, Western Ontario, and McMaster Universities Osteoarthritis Index (WOMAC) [27] satisfaction and preference. The radiological results included the alignment of the limb (femorotibial angle), the patellar tilt angle, the posterior condylar offset, or radiolucent lines.

### Quality assessment

A risk-of-bias evaluation was performed using the Cochrane Collaboration tool [28]. Seven domain-based evaluations related to risk of bias were performed, including blinding of the participants and personnel (performance bias), evaluation for random sequence generation (selection bias), blinding of the assessors (defection bias), allocation concealment (selection bias), selective reporting (reporting bias), incomplete outcome data (attrition bias), and other biases. The overall quality of each study was evaluated as a “low risk of bias”, a “high risk of bias”, or an “unclear risk of bias”.

A modified Jadad score was used for the quality evaluation of PRCTS including data analysis, blinding, randomization, withdrawal, adverse reactions, and inclusion criteria. Low-quality studies scored from 0 to 3 and high-quality studies scored from 4 to 8.

### Statistical analysis

Descriptive statistics, such as means, ranges, and measures of variance (standard deviations, 95% confidence intervals (CI)), are presented where applicable. No meta-analysis was performed.

## Results

**Figure 1:**
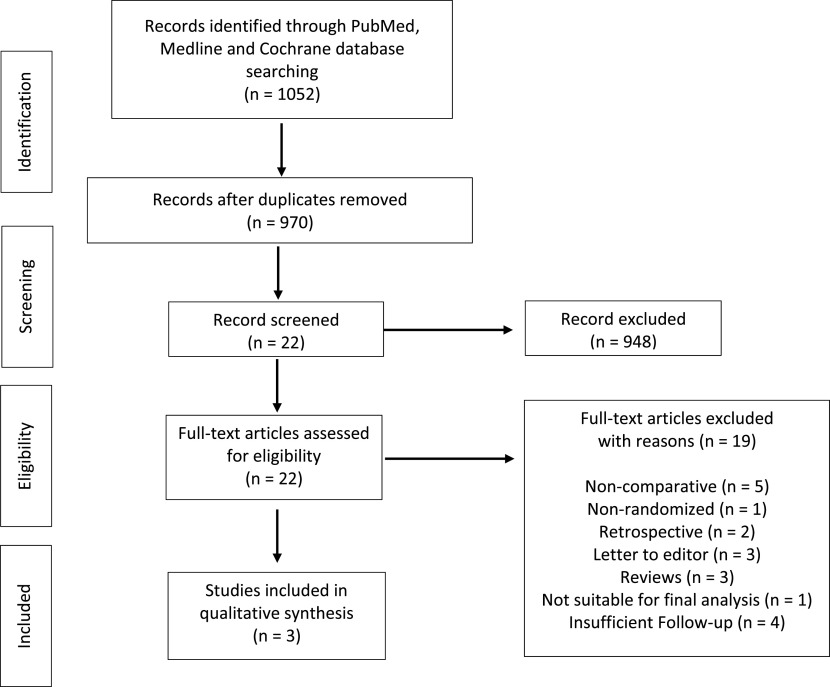
Flow chart.

The selection procedure is shown in Figure 1. A total of 1052 studies were identified by using our primary and secondary search strategy. After exclusion of duplicate studies, a total of 970 studies remained for further screening. Examination of title/abstracts excluded 948 records, and a further 19 were excluded after the studies were examined closely. Thus, three PRCTs were included [29–31] and were published between 2010 and 2012. Characteristics of the studies included are reported in Table 1.

**Table 1:** Characteristic of the studies.

Studies	Location	Study design	Minimum follow-up (m)	Sample size		Mean age	BMI (mean)	Prosthesis design	Clinical measurements	Radiological measurements	Other measurements
				GS	Conv						
Kim et al. [29]	South Korea	PRCT	37	138	138	71.2	27.3	*GS*: NexGen gender-specific CR-flex, cemented, all PR	WOMAC, KSS, ROM, pain, satisfaction, preference	Radiographic outcomes	Complications
								*Conv*: NexGen standard CR-flex, cemented, all PR			
Kim et al. [30]	South Korea	PRCT	24	85	85	69.7	27.1	*GS*: NexGen gender-specific posterior cruciate-substituting flex, cemented, all PR	WOMAC, HSS, KSS, ROM, pain, satisfaction, preference	Radiographic outcomes	Complications
								*Conv*: NexGen standard posterior cruciate-substituting flex, cemented, all PR			
Song et al. [31]	South Korea	PRCT	24	46	46	68.8	26.8	*GS*: NexGen gender-specific CR-flex, cemented, no PR	WOMAC, HSS, ROM, preference	Radiographic outcomes	
								*Conv*: NexGen standard CR-flex, cemented, no PR			

PRCT: prospective randomized controlled trial, GS: gender-specific, Conv: conventional, CR: cruciate-retaining, PR: patella resurfacing, WOMAC: Western Ontario and McMaster Universities Osteoarthritis Index, KSS: knee society score, ROM: range of motion, HSS: Hospital for Special Surgery knee score.

### Risk of bias and quality of evidence

**Figure 2:**
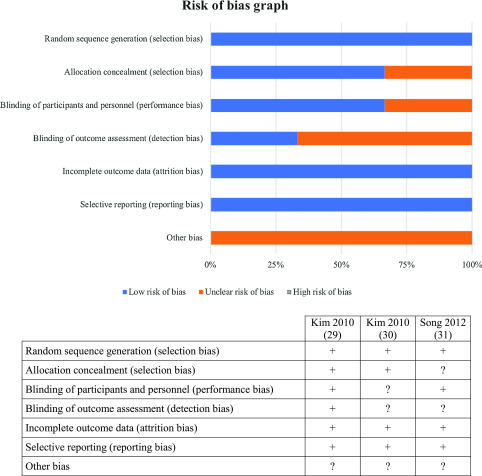
Risk of bias graph; “+ or plus” indicates a low risk of bias; “− or minus” indicates a high risk of bias; and “? or question mark” indicates unclear of unknown risk of bias.

The results of the risk-of-bias assessment of the studies are reported in Figure 2. There was an unclear risk of bias in two studies in blinding the outcome assessment (detection bias). There was an unclear risk of bias in one study in allocation concealment (selection bias). There was an unclear risk of bias in one study in blinding the participants and personnel (performance bias). There was an unclear risk of bias for the category “other bias” in the three studies. We did not find any other apparent bias in any of the included studies. The quality evaluation scores of the studies are shown in Table 2. Two studies included in this systematic review were given scores of 5 and one study had a score of 7. Thus, after examination, the three included studies were of very high quality and had a low risk of bias.

**Table 2:** Modified Jadad-score.

	Kim et al. [29]	Kim et al. [30]	Song et al. [31]
Was the study described as randomized?	Yes	Yes	Yes
Was the method of randomization appropriate?	Yes	Yes	No
Was the study described as blinded?	No	No	Yes
Was the method of blinding appropriate?	No	No	Yes
Was there a description of withdrawals or dropouts?	Yes	Yes	Yes
Was there a clear description of the inclusion/exclusion criteria?	Yes	Yes	Yes
Was the method used to assess adverse effects described?	No	No	Yes
Was the method of statistical analysis described?	Yes	Yes	Yes
Total score	5	5	7

### Clinical results

For the three studies, clinical results were significantly improved postoperatively in both groups (gender-specific and conventional) without showing any significant difference between groups for all scores [29–31]. All clinical results are reported in Table 3. Two studies reported postoperative complication rate and no significant difference was found between GS and conventional groups at the last follow-up.

**Table 3:** Clinical results at the last follow up (SD = standard deviation).

Studies	Clinical assessment	Gender-specific prosthesis mean ± SD (range)	Conventional prosthesis mean ± SD (range)	*p*-value
Kim et al. [29]	KSS knee	93 (70–100)	94 (70–100)	0.69
	KSS functional	84 (60–100)	83 (60–100)	0.322
	Flexion	124 (85–140)	126 (85–140)	0.002
	Pain	46.6 (20–50)	46.8 (20–50)	0.667
	Satisfaction	7.9 ± 2.1	8.1 ± 1.9	0.187
	Preference	14 (10.1%)	12 (8.7%)	>0.05
	Complications	1	1	>0.05
Kim et al. [30]	WOMAC	35.7 (5–61)	36.6 (4–69)	0.189
	HSS	91.2 (77–100)	90.7 (84–100)	0.252
	KSS knee	96.5 (83–100)	95.5 (81–100)	0.424
	KSS functional	84.8 (60–100)	84.8 (60–100)	>0.05
	Flexion	126 (85–140)	125 (80–140)	0.739
	Pain	46.3 (40–50)	45.1 (40–50)	0.838
	Satisfaction	8.1 ± 1.9	8.3 ± 1.7	0.783
	Preference	6 (7%)	8 (9%)	>0.05
	Complications	1	1	>0.05
Song et al. [31]	WOMAC	31.6 ± 8.5 (24–52)	32.6 ± 9.2 (24–58)	0.58
	HSS	92.7 ± 8.0 (75–100)	92.1 ± 8.7 (67–100)	0.75
	Flexion	131.1 ± 9.2	133.7 ± 19.2	0.16
	Preference	10 (10.9%)	7 (7.6%)	0.59

KSS: knee society score, WOMAC: Western Ontario and McMaster Universities Osteoarthritis Index, HSS: Hospital for Special Surgery knee score.

### Radiological results

Two studies reported radiolucent lines of <1 mm in their study without any significant differences between groups. Two studies reported prostheses overhang. Gender-specific TKA significantly reduced the overhang rate compared to conventional TKA. Indeed, Kim et al. [29] showed in the group with conventional implants 14 knees had an overhang (mean, 1.7 mm; range, 1–4 mm), and 24 knees had an under-coverage (mean, 1.6 mm; range, 1–5 mm); and in the group with a gender-specific implant, 123 knees had an under-coverage (mean, 3.7 mm; range, 1–11 mm). Kim et al. [30], in another study, showed in the group with conventional implants 10 knees had an overhang (mean, 1.4 ± 0.7 mm; range, 1–3 mm), and 24 knees had an under-coverage (mean, 1.1 ± 0.3 mm; range, 1–2 mm); and in the group with a gender-specific implant, 71 knees had an under-coverage (mean, 2.8 ± 1.3 mm; range, 1–7 mm). No significant difference was found between both groups for patellar tilt angle. All radiographic results are reported in Table 4.

**Table 4:** Radiological results at the last follow up (SD = Standard deviation).

Studies	Radiological assessment	Gender-specific prosthesis mean ± SD (range)	Conventional prosthesis mean ± SD (range)	*p*-value
Kim et al. [29]	HKA (°)	186.6 (182 to 187)	186.3 (183 to 187)	0.970
	TS (°)	7 (2 to 12)	7.6 (1 to 14)	0.492
	PCO (mm)	0.3 (0 to 18)	−0.3 (−2 to 0)	0.148
	PTA (°)	3.8 ± 1.2	3.3 ± 1.7	0.919
	Radiolucent line	11 (8.0%)	12 (8.7%)	>0.05
	Overhang	0 (0%)	14 (10.1%)	<0.001
	Under-coverage	123 (89%)	44 (31.9%)	<0.001
Kim et al. [30]	HKA (°)	186.4 (181.5 to 188)	185.8 (182 to 187)	0.901
	TS (°)	7 (−2 to 14)	7.6 (1 to 12)	0.699
	PCO (mm)	0.3 (0 to 1)	−0.5 (−2 to −1)	0.151
	PTA (°)	3.6 ± 1.6	3.8 ± 1.3	0.873
	Radiolucent line	6 (7%)	17 (8%)	1.0
	Overhang	0 (0%)	10 (12%)	0.0011
	Under-coverage	71 (84%)	24 (28%)	<0.001
Song et al. [31]	HKA (°)	185.96 ± 2.2	185.7 ± 2.1	0.54
	TS (°)	7.6 ± 3.1	6.2 ± 2.5	0.08
	PCO (mm)	1.4 ± 3.2	0.7 ± 4.0	0.05
	ACO (mm)	1.3 ± 2.9	0.2 ± 1.5	0.08
	PTA (°)	6.0 ± 3.8	7.7 ± 4.4	0.83

HKA: hip-knee-ankle angle, TS: tibial slope, PCO: posterior condylar offset, PTA: patellar tilt angle, ACO: anterior condylar offset.

## Discussion

This review is a recent update of all comparative studies between GS implants and standard unisex implants with a minimum follow-up of two years. All studies included were level I and therefore of very high quality. The principal findings of this systematic review were as follows: the clinical results of GS TKA were similar to those of conventional TKA and a significantly reduced overhang rate was found for GS prostheses compared to conventional implants. Our study is more strict on inclusion criteria compared to previous literature review on this topic and may be more robust in its conclusion.

GS knee prostheses were introduced based on the assumption that TKA outcomes might be inferior in women compared to men when using standard prostheses, although one study reported that women achieved similar or even better results in terms of pain, satisfaction, range of motion, satisfaction, and implant survival when standard implants were used [32]. Standard unisex implants may lead to overhang, which can be resolved by using GS implants for women, as they have a different distal femoral aspect ratio and a higher *Q*-angle. Indeed, using GS prosthesis should potentially reduce the incidence of overhang and therefore, in theory, reduce post-operative medial and lateral knee pain due to soft-tissue irritation. Mahoney and Kinsey [21] showed that significantly more women had lateral and medial overhang than men when a conventional prosthesis was used. They also found that an overhang of the femoral component ≥3 mm was related to post-operative knee pain. When a GS prosthesis was implanted in women, Clarke and Hentz [18] found a decrease in the occurrence of overhang (17% vs. 5%). In this systematic review, two studies reported a significantly reduced femoral component overhang in the GS group (0 mm vs*.* 1.4–1.7 mm) without any significant difference in clinical outcomes between groups. The observed overhang, which was less than 3 mm, may explain the absence of significant clinical difference between groups by being insufficient. Furthermore, it is important to note that in the GS group, a higher incidence of underhang was observed, which exposed more cancellous bone and could be a source of higher perioperative blood loss, and may induce increased osteolysis from wear debris at longer follow-up [10, 29, 30].

Overstuffing of the anterior knee compartment may be associated with reduce ROM and pain. Women having a less prominent native anterior femoral condyle, using a standard unisex TKA could possibly lead to overstuffing [14, 33]. Despite the fact that reduced height of the anterior femoral implant flange and the deeper trochlear groove improve patellar tracking in the GS design and help prevent overstuffing of the patellofemoral joint, we did not find any significant difference in postoperative pain and ROM between groups.

On the other hand, several studies refuted the assumption that women have worse outcomes than men using standard unisex TKA designs [3, 32–34]. Indeed, some studies showed similar, or even better, results between women and men [5, 32, 34]. Merchant et al. [32], in a systematic review, reported no evidence of anatomical differences between male and female knees that would justify a female-specific design. The anatomical differences between female and male knees can be explained by the smaller size and height of women on average, not by their gender. Bellemans et al. [35] reported that the shape of the knee is not only dependent on gender, but also on the morphotype of the patient. Piriou et al. [36] showed similar findings in their study, with findings that the distal femoral epiphysis was only related to femoral length, independent of gender.

Several limitations should be discussed. Firstly, we only found three relevant studies that compared GS with conventional TKA with a minimum follow-up of two years. Thus, the follow-up period was short. However, earlier studies have revealed that the range of movement and satisfaction reaches a plateau beyond one year [37, 38]. Secondly, our data analyzed only a single implant design (Zimmer Gender). These results may not be applied to other TKA designs. Finally, all studies included were already included in previous literature reviews and no new papers or data since 2012 were included in our analysis as nothing has been published since.

## Conclusion

In our systematic review, despite less femoral prosthesis overhang rate with GS prostheses, we conclude that gender-specific implants in female total knee replacements showed no clinical benefit over standard unisex implants. Good clinical results with significant improvement were observed with both designs. There is a notable absence of new studies on this subject in recent years, and further research needs to be performed using various gender-specific implant designs to further define the role of gender-specific implants.
